# A novel single robot image shadow detection method based on convolutional block attention module and unsupervised learning network

**DOI:** 10.3389/fnbot.2022.1059497

**Published:** 2022-11-08

**Authors:** Jun Zhang, Junjun Liu

**Affiliations:** ^1^Office of Academic Affairs, Zhengzhou University of Science and Technology, Zhengzhou, China; ^2^College of Information Engineering, Zhengzhou University of Science and Technology, Zhengzhou, China

**Keywords:** robot image shadow detection, hierarchical domain adaptation strategy, boundary adversarial branch, unsupervised learning, convolutional block attention module

## Abstract

Shadow detection plays a very important role in image processing. Although many algorithms have been proposed in different environments, it is still a challenging task to detect shadows in natural scenes. In this paper, we propose a convolutional block attention module (CBAM) and unsupervised domain adaptation adversarial learning network for single image shadow detection. The new method mainly contains three steps. Firstly, in order to reduce the data deviation between the domains, the hierarchical domain adaptation strategy is adopted to calibrate the feature distribution from low level to high level between the source domain and the target domain. Secondly, in order to enhance the soft shadow detection ability of the model, the boundary adversarial branch is proposed to obtain structured shadow boundary. Meanwhile, a CBAM is added in the model to reduce the correlation between different semantic information. Thirdly, the entropy adversarial branch is combined to further suppress the high uncertainty at the boundary of the prediction results, and it obtains the smooth and accurate shadow boundary. Finally, we conduct abundant experiments on public datasets, the RMSE has the lowest values with 9.6 and BER with 6.6 on ISTD dataset, the results show that the proposed shadow detection method has better edge structure compared with the existing deep learning detection methods.

## 1. Introduction

Shadows exist in most scenes in our daily life, which are shielded by light sources. Shadows can preserve important information about dynamic scene and objects, such as detection of buildings and vegetation areas, and detection of clouds through shadows in satellite images. On the other hand, shadows are also a major source of error and uncertainty (Shoulin et al., 2018; Sun et al., 2019; Yuan et al., 2020). For example, shadows may be wrongly labeled as targets in dynamic target tracking tasks. Therefore, shadow detection in images can significantly improve the performance of many visual tasks. The shape and brightness of the shadow depends on the intensity, direction, color of the light source, and the geometry and albedo of the shade. Shadows can be divided into hard shadows and soft shadows based on their intensity. Hard shadows have relatively clear shadow boundaries, while soft shadows are often generated when the light source intensity is low, and the shadow boundaries are blurred. Most existing shadow detection methods are usually limited to hard shadow detection. Compared with video shadow detection, single image shadow detection is more challenging because of the lack of relevant information before and after frames (Sun et al., 2018).

Most traditional shadow detection methods are based on the fact that the brightness of the shadow pixel is different from that of the non-shadow pixel (Vicente et al., 2018). In addition, Wang et al. (2018) firstly divided images into multiple image blocks based on statistical learning method, and then classified these blocks using Least Squares Support Vectors Machine (LSSVM) to obtain shadow detection results. In recent years, many methods based on deep learning have quickly become the benchmark due to their good effects and calculation efficiency. For example, Khan et al. (2016) combined Conditional Random Field (CRF) and convolutional neural network (CNN) to extract the local features of shadow pixels in the image. In Yago Vicente et al. (2016), a stacked convolutional neural network (Stacked CNN) was proposed based on a large-scale shadow detection data set. It allowed one CNN with learned semantic features to train another CNN and refined the details of the shadow areas. Recently, Nguyen et al. (2017) proposed a novel shadow detection method based on Conditional Generative Adversarial Network (CGAN), which benefited from special sensitivity factors and adversarial learning framework, which could obtain relatively accurate shadow mask. Based on the idea of adversarial learning, Le et al. (2018) trained a shadow image attenuator to generate additional challenging image data to enhance the robustness of shadow detection. Wang et al. (2018) proposed the Stacked Conditional Generative Adversarial Network (ST-CGAN), which used two CGAN for shadow detection task and shadow removal task, respectively. Mohajerani and Saeedi (2018) preserved the global semantic features of shadows by changing the internal connection of the network to enhance the ability of shadow detection based on U-Net13.

The above methods can be roughly divided into the traditional machine learning methods based on custom features and the feature learning methods based on deep learning (Ji et al., 2021; Ma et al., 2021; Shafiq and Gu, 2022). Due to the lack of prior information of light source or occlusion, traditional machine learning methods based on custom features often lack robust custom features and cannot accurately understand shadows. Through many rich experiments, although many deep learning methods are more accurate than traditional methods, they usually only have good results on homologous test sets. In addition, most shadow images in common data sets are strong shadow images captured by artificial occlusion (Kamnitsas et al., 2017; Shafiq et al., 2020b; Hatamizadeh et al., 2022). However, the shapes and scenes of shadows are not limited to such shadows, such as shadows on buildings or soft shadows cast when the light source is not strong enough. They do not have clear shadow boundaries. Deep learning methods used to detect shadow images in these target domains (target datasets) often only produce incomplete and jagged shadow detection results.

To solve the above problems, our research goal is that a novel unsupervised domain adaptation adversarial learning network for single image shadow detection is proposed in this paper. The model is trained by supervised learning on the source data set. But for the unused target data set, the complex artificial labeling process is considered to make the model have the same performance on the target data set, and enhance the robustness of the model. Specifically, in the process of feature extraction, the multi-layer feature domain adaptation strategy is combined to minimize the data deviation between the source domain and the target domain. Secondly, the boundary adversarial branch is proposed, and the boundary generator and boundary discriminator are used to strengthen the boundary structure of soft shadow detection results. Finally, entropy adversarial branch is introduced to reduce the uncertainty of the shadow boundary region in the shadow image, and a smooth and accurate shadow mask is obtained.

This paper is organized as follows. Section 2 detailed introduces the proposed domain adaptation adversarial learning network for shadow detection. We conduct rich experiments in section 3. There is a conclusion in section 4.

## 2. Proposed unsupervised learning network

**Figure 1:**
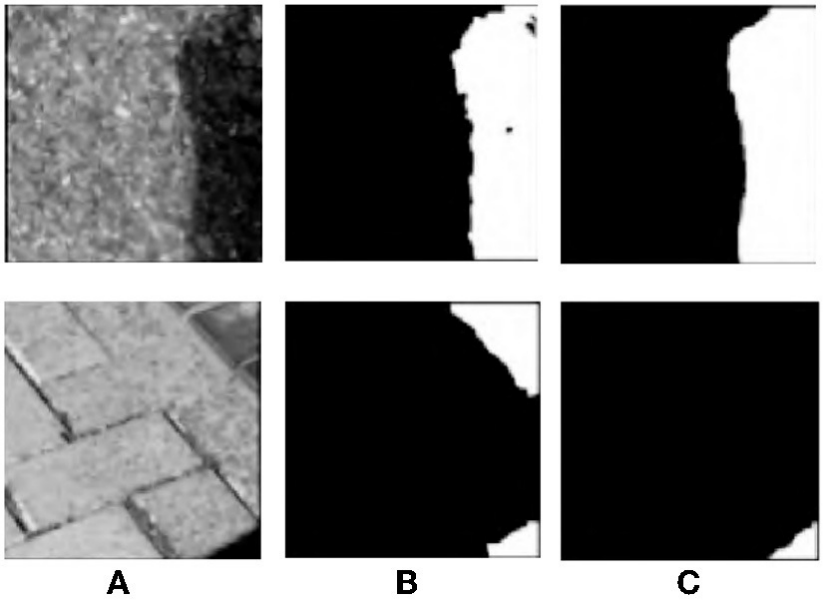
Analysis of cross-domain discrepancy. First row: Source data set. Second row: Target data set. First column: shadow image **(A)**. Second column: CGAN method **(B)**. Third column: proposed method **(C)**.

Different “domains” are actually different data sets. The process of domain adaptation aims to make a model adapt to multiple different domains, so that the model can be better generalized to other data sets. Many supervised deep learning methods can bring significant performance improvement for shadow automatic detection, but due to cross-domain discrepancy (Shafiq et al., 2020a), the model cannot get satisfactory results on the target data set. As shown in Figure 1, through many experiments and analysis, the deep network trained on source data set ISTD can usually only generate relatively accurate shadow results for its homologous test images. When applied to the target data set SBU, the boundary structure of shadow detection results is poor, as shown in Figure 1B. The proposed model not only performs well on the source data set, but also has good detection capability on the target data set, as shown in Figure 1C. Compared with these methods, when facing a new data set, the proposed method is no longer need the tedious manual labeling work as training data to provide the corresponding shadow labeled data, it uses the unsupervised learning aiming to make the model easily realize the domain adapt to get accurate shadow detection results for new data sets.

**Figure 2:**
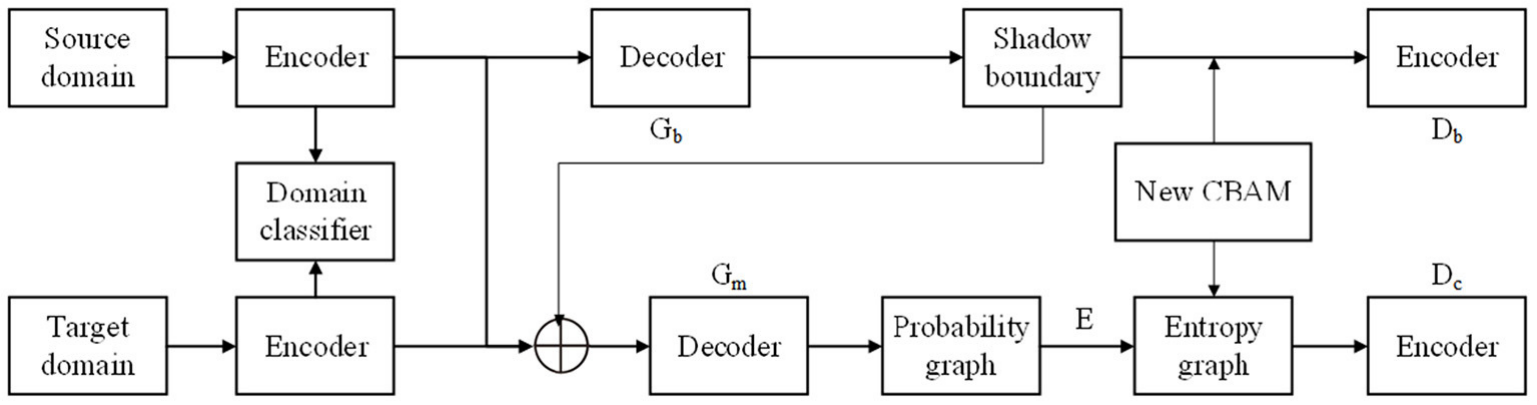
Proposed single robot image shadow detection.

The proposed shadow detection framework is shown in Figure 2. For the shadow images in the source domain and the target domain, a separate feature extraction channel is firstly adopted, and the domain discriminator is used to judge the domain label of the current feature from the low level to the high level. Then, two generative adversarial branches are constructed. The boundary adversarial branch is used to enhance the detection ability of soft shadow image in the target dataset (Lee et al., 2022). The entropy adversarial branch can further suppress the uncertainty at the boundary of the shadow, so that a smooth and accurate shadow mask can be obtained. With the objective function and special network connection, the two tasks are mutually constrained and promoted to achieve accurate cross-domain shadow detection.

### 2.1. Hierarchical feature extraction method

The traditional domain adaptation model only corrects the feature distribution between different domains in the last convolution layer to realize the whole local adaptation (Chen et al., 2018). However, this method ignores the importance of low-level features, and makes some domain-sensitive local features weaken the generalization ability of the domain adaptation model. Because of the non-transferable layer, a single domain classifier is difficult to eliminate the data deviation between the source domain and the target domain. Inspired by Shafiq et al. (2020c) and Zhang et al. (2020), shadow images are taken as input in the source domain and target domain. In the process of image encoding, each convolution layer in the encoder has a corresponding feature graph. It extracts the output feature graphs of multiple middle layers in the encoder. The corresponding image domain classifier is constructed on each convolution layer between the encoders of the source domain and the target domain to promote the feature matching in the middle layer. The aim is to make two different encoders still have similar feature extraction process under different data sets to achieve the purpose of domain adaptation. The objective function is shown in Equation (1):


(1) $$\begin{array}{l} L_{M}=-\underset{i,k,o,p}{\sum }\left[D_{i}lnf_{k}\left(\Phi_{i,k}^{o,p}\right)+\left(1-D_{i}\right)ln\left(1-f_{k}\left(\Phi_{i,k}^{o,p}\right)\right)\right] \end{array}$$

where *D*_*i*_ is the domain label of the *i* − *th* image. $\Phi_{i,k}^{o,p}$ represents the feature graph activation value of the pixel at the *k* − *th* layer with coordinate (*o, p*) in the *i* − *th* image. *f*_*k*_ is the corresponding domain classifier.

**Figure 3:**
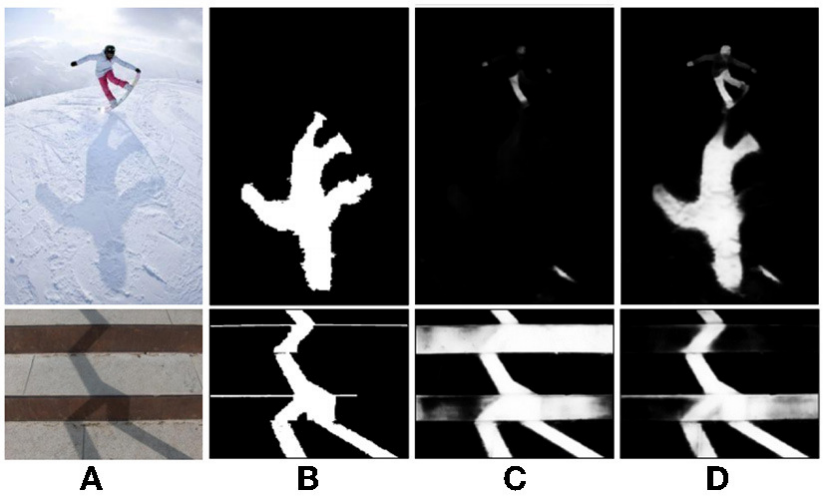
Effect of hierarchical domain adaptation on shadow detection. First column **(A)**, second column **(B)**, third column **(C)**, and fourth column **(D)** are shadow image, GT, global domain adaptation, hierarchical domain adaptation, respectively.

Hierarchical domain adaptation ensures that the intermediate features between the two domains have similar distribution, thus enhancing the robustness of the adaptation model. In the process of shadow detection, eliminating the data deviation between domains can improve the accuracy of shadow detection on the target data set. As shown in Figure 3, Figure 3A is the shadow image in the target domain. Figure 3B is the label data (ground truth). Figure 3C shows the shadow detection results with the global domain adaptation. Figure 3D shows the shadow detection results with the hierarchical domain adaptation. Compared with Figures 3C,D, the model obtains a better generalization after the hierarchical domain adaptation feature extraction, and has a more accurate detection ability for the text with different colors adjacent to the shadow in the image.

### 2.2. A hybrid domain attention mechanism with CBAM

For computer vision tasks, the attention mechanism plays the role of generating weights for each pixel of the image. Ideally, the weight of foreground pixel will increase and the weight of background pixel will decrease gradually. Through the widening of the weight gap, the effect of different semantic separation will be achieved.

Convolutional Block Attention Module (CBAM) is a reliable attention mechanism algorithm in computer vision tasks, which has a simple algorithm structure and considerable practical effect. Convolutional block attention module combines the space and channel of CNN to generate respective attention for images and feature maps of different attention domains, and guides the model to distinguish semantic information more efficiently.

Convolutional block attention module is composed of spatial domain attention generation module and channel domain generation module, and the two modules need to be combined by weighted sum operation. Where, the space domain generation module can be expressed as:


(2) $$\begin{array}{l} F_{Avg}^{S}=AvgPool\left(F\right) \end{array}$$

(3) $$\begin{array}{l} F_{Max}^{S}=MaxPool\left(F\right) \end{array}$$

(4) $$\begin{array}{l} M_{S}F=Sigmoid\left(f^{7\times 7}\left(F_{Avg}^{S}+F_{Max}^{S}\right)\right) \end{array}$$

**Figure 4:**
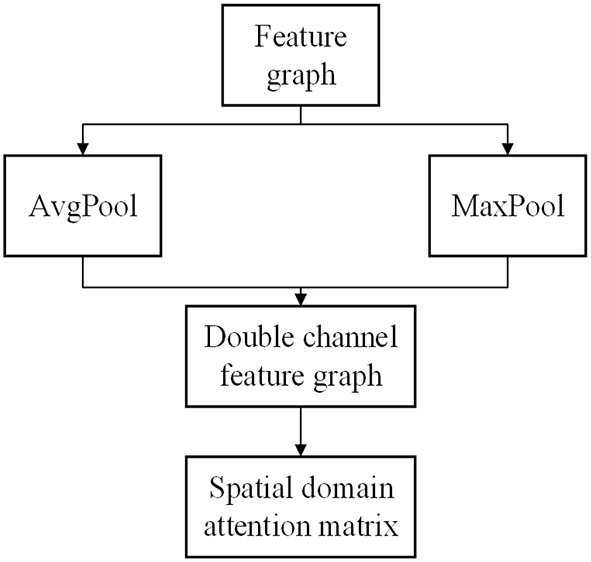
CBAM.

According to the feature map *F* output by the CNN, the global average pooling and global maximum pooling operations of the feature map are carried out simultaneously. Then, the results of the two pooling methods are connected based on channels, and a convolution network with the number of target channels is 1 and the convolution kernel is 7 × 7 is input. The number of channels is reduced to 1 without changing the length and width of the feature map. Then the activation function Sigmoid is used to transform the output into nonlinear data, and the spatial domain attention matrix *M*_*s*_(*F*) is obtained. Figure 4 shows the spatial domain generation module of CBAM.

The channel domain generation module can be expressed as:


(5) $$\begin{array}{l} F_{Avg}^{C}=MLP\left(AvgPool\left(F\right)\right) \end{array}$$

(6) $$\begin{array}{l} F_{Max}^{C}=MLP\left(MaxPool\left(F\right)\right) \end{array}$$

(7) $$M_{c}F=Sigmoid\left(F_{Avg}^{C}+F_{Max}^{C})\right)$$

In the attention module of channel domain, the average pooling and maximum pooling operations based on channel are carried out synchronously in the feature map *F*. Then the results of the two operations are respectively input into the same multi-layer perceptron, and the two vectors are directly added together. It inputs Sigmoid activation function, and outputs channel domain attention matrix *M*_*c*_(*F*).

### 2.3. Boundary feature analysis

**Figure 5:**
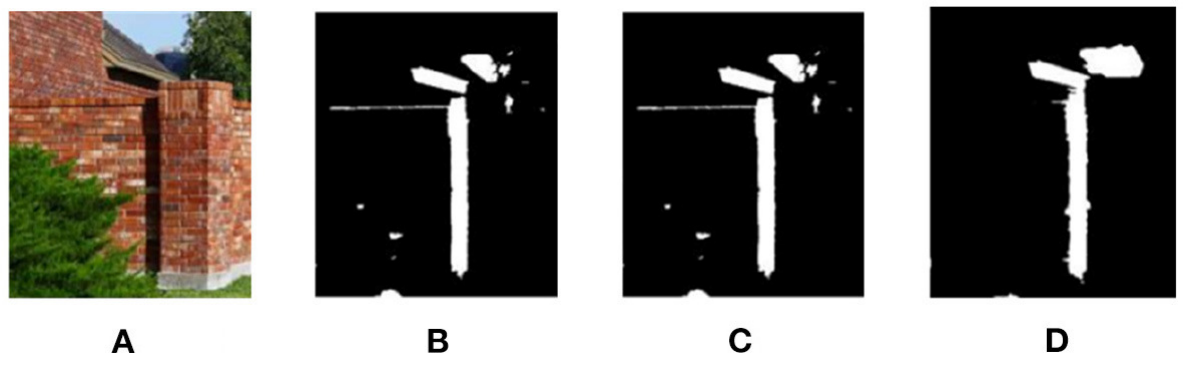
Analysis of boundary adversarial branch. **(A)** Shadow image, **(B)** before boundary adversarial, **(C)** after boundary adversarial, and **(D)** detection result.

The existing shadow detection data sets lack the soft shadow images with rich scenes because of the single acquisition method (using various shielders under the strong light source). Affected by the intensity of light source, soft shadow image does not have clear shadow boundary. However, many existing deep learning methods are unable to obtain good detection results on soft shadow images. As shown in Figure 5, for soft shadow images in the target data set, the detection result of boundary structure cannot be obtained only by correcting feature distribution, as shown in Figure 5B.

In order to solve the above problems, the boundary adversarial branch model is constructed to predict the boundary structure results in the target data set. Boundary adversarial branch is designed to generate the shadow boundary image as shown Figure 5C. Then, based on the principle of adversarial learning, the discriminator is used to further improve the quality of the generated image. With the initial positioning of the shadow boundary, the subsequent shadow detection results will have more boundary structure and ultimately improve the detection ability of soft shadow.

Assume that the source data set is *S*. Its label data is the ground truth mask *y*_*s*_. The target dataset *T* has no labeled data. Firstly, the generator *G*_*b*_ fits the shadow boundary in the image and generates boundary prediction results *G*_*b*_(*x*_*s*_) and *G*_*b*_(*x*_*t*_) for the light source shadow image *x*_*s*_ and target shadow image *x*_*t*_, respectively. The visualization is shown in Figure 5C. Secondly, the discriminator *D*_*b*_ is designed to determine whether the boundary comes from the source or the target dataset. With the boundary adversarial branch, for the soft shadow image in the target domain, it can accurately identify the shadow region, as shown in Figure 5D.

For the source domain data set and target domain data set with domain label, boundary discriminator *D*_*b*_ judges and punishes *G*_*b*_(*x*_*s*_) and *G*_*b*_(*x*_*t*_), respectively, as shown in Equation (8):


(8) $$\begin{array}{l} L_{D_{b}}=\frac{1}{N}\underset{x_{t}\in S}{\sum }L_{B}\left(G_{b}\left(x_{s}\right),1\right)+\frac{1}{M}\underset{x_{t}\in T}{\sum }L_{B}\left(G_{b}\left(x_{t}\right),0\right) \end{array}$$

where *L*_*B*_ is binary cross entropy loss, which is defined as *L*_*B*_(ŷ, *y*) = −(*ylnŷ* + (1 − *y*)*ln*(1 − ŷ)); *N* and *M* are the number of images in the source data set and the target data set, respectively.

The loss function *L*_*G*_*b*__ of the generator is a weighted combination of the mean absolute error loss term on the source data set and the adversarial loss term on the target data set, as shown in Equation (9):


(9) $$L_{G_{b}}=\frac{1}{N}\sum_{x_{s}\in S}||y_{x_{s}}^{b}-(G_{b}(x_{s})|{|}_{1}+\lambda_{1}\frac{1}{M}\sum_{x_{s}\in T}L_{B}(G_{b}(x_{s}),1)$$

where $y_{x_{s}}^{b}$ is the shadow boundary label image in the source data set.

### 2.4. Entropy mask prediction

After the boundary adversarial branch, using the additional shadow mask generator directly generates zigzag shadow detection boundaries for the target dataset (Figure 5B). Inspired by Vu et al. (2019), the shadow mask results have a high entropy value (uncertainty) in the region near the shadow boundary, which will lead to the zigzag boundary phenomenon.

In order to suppress the uncertain prediction results, the entropy adversarial branch first generates the shadow probability map for the shadow image. Based on the probability map, the Shannon entropy is used to transform the probability map into the entropy map. Entropy maps of the target domain and source domain are forced to be as similar as possible, so as to reduce the effect difference between the model on the target and source data sets. Finally, the quality of the generated image is improved by the idea of adversarial learning. The high entropy value in the entropy graph should only be around the shadow boundary. The reasonable entropy distribution corresponds to the shadow detection results with smooth boundary.

Mask generator *G*_*m*_ generates mask prediction results *G*_*m*_(*x*_*s*_) and *G*_*m*_(*x*_*t*_) for source and target images, respectively. Given the mask prediction result *p* of input image *x*, Shannon entropy can be used to calculate the entropy graph, as shown in Equation (10):


(10) $$\begin{array}{l} E\left(x\right)=p\times log\left(p\right) \end{array}$$

Entropy discriminator *D*_*e*_ aims to calibrate the distribution of *E*(*x*_*s*_) and *E*(*x*_*t*_). Similar to the boundary-driven adversarial learning, the entropy discriminator *D*_*e*_ determines whether the entropy graph comes from the source domain or the target domain. Its objective function is shown in Equation (11):


(11) $$\begin{array}{l} L_{D_{e}}=\frac{1}{N}\underset{x_{t}\in S}{\sum }L_{B}\left(E\left(x_{s}\right),1\right)+\frac{1}{M}\underset{x_{t}\in T}{\sum }L_{B}\left(E\left(x_{t}\right),0\right) \end{array}$$

The loss function *L*_*G*_*m*__ of the generator is a weighted combination of the pixel-level cross entropy loss on the source data set and the adversarial loss item on the target data set, as shown in Equation (12):


(12) $$\begin{array}{l} L_{G_{m}}=-\frac{1}{N}\underset{x_{s}\in S}{\sum }\left(y_{x_{s}}^{m}\cdot ln\left(G_{m}\left(x_{s}\right)\right)+\left(1-y_{x_{s}}^{m}\right)\cdot ln\left(1-G_{m}\left(x_{s}\right)\right)\right)\\ \;+\lambda_{2}\frac{1}{M}\underset{x_{s}\in T}{\sum }L_{B}\left(E\left(x_{t}\right),1\right) \end{array}$$

where $y_{x_{s}}^{m}$ is the shadow mask label image.

### 2.5. Shadow removal

Firstly, the coherence block matching strategy is used to find the best matching non-shaded region block for each region block in the shaded region. Then, local illumination propagation and global illumination optimization were performed for each matching shaded and non-shaded area pair. Finally, the shadow boundary is processed to get the final result.

**A. Local illumination propagation**

The shadow area is modeled and combined with the illumination propagation algorithm, the ratio of direct light and indirect light is calculated to obtain the video without shadow:


(13) $$\begin{array}{l} I_{i}=\left(k_{i}L_{d}+L_{e}\right)R_{i} \end{array}$$

where *I*_*i*_ is the *i* − *th* pixel of the image in RGB space. *k*_*i*_ ∈ [0, 1] is the degree of direct illumination of the pixel. Both *L*_*d*_ and *L*_*e*_ are vectors of scale 3, representing the intensity of direct light and ambient light. *R*_*i*_ is the reflectance of the pixel, which is also a three-dimensional vector, and each dimension corresponds to a color channel of the RGB image. Equation (13) indicates that the pixel value of a pixel is obtained by the interaction of direct light and ambient light and multiplied by the reflectance of the pixel. The state of direct light in the image can be divided into three situations, namely, shaded area, non-shaded area, and semi-shaded area. When *k*_*i*_ = 0, the pixel is not affected by direct light and belongs to the shadow area. When *k*_*i*_ = 1, direct light completely acts on the pixel, and the pixel belongs to the non-shadow region. When *k*_*i*_ ∈ (0, 1) is the shaded transition region.

Then for a pixel with the shadow removed, the relationship between its pixel value and the pixel value of the shadow pixel can be simplified as Equation (14):


(14) $$\begin{array}{l} I_{i}^{\prime }=\left(L_{d}+L_{e}\right)R_{i}=\frac{r+1}{k_{i}r+1}I_{i} \end{array}$$

where $r=\frac{L_{d}}{L_{e}}$ is the ratio of direct light to ambient light. *I*_*i*_ is the RGB value of the *i*-th pixel of the original image.

**B. Global shadow removal**

Although the local illumination propagation operation can remove the shadow in the shadow area block, it can not get the spatio-temporal coherent shadow free video result. After the local illumination propagation, the global shadow removal method should be used to make up for this deficiency. In order to obtain spatially coherent unshaded images $V_{s}^{\prime }$, a weighted average method is proposed to recover the unshaded values of pixels in overlapping areas in the following equation.


(15) $$\begin{array}{l} V_{p}^{\prime }=\underset{S_{i}\in N_{s}\left(p\right)}{\sum }V_{s_{i}}^{\prime }\left(p\right)w_{i}/\underset{i}{\sum }w_{i} \end{array}$$

where *N*_*s*_(*p*) is the block containing pixel *p*. *w*_*i*_ = *dist*(*i, j*) is the similarity distance between block *s*_*i*_ and its corresponding block *L*_*j*_. Where *N*_*L*_(*p*) is the block set formed by the nearest *n* *N*_*s*_(*p*) corresponding to the bright region. $V_{p}^{\prime }$ is the result of local illumination propagation of pixel *p* through both block *S*_*i*_ and block *L*_*i*_. In summary, the pixel value of pixel *p* in the overlapping area is obtained by the weighted average of multiple blocks containing pixel *p*.

Using the above global optimization technique, spatially smooth shadow-free results can be obtained within the shadow. Using weighted average method to calculate the shadow free value of overlapping pixels can avoid or greatly reduce the fuzzy artifacts in the overlapping area. Minimizing the objective function ensures that the results are consistent in time.


(16) $$\begin{array}{l} E(V^{f})=\sum_{p\in S}=\sum_{p\in S}\phi ({\left[V^{f}(p)-{V^{\prime }}_{s}(p+u(p))\right]}^{2})+\phi ([V^{f}(p)\\ \;-{V^{\prime }}_{s}(p+v(p)){]}^{2}) \end{array}$$

where *V*^*f*^ is the final shadow-free result. *S* represents the shaded area. *u*(*p*) and *v*(*p*) are the forward and backward optical flow direction of pixel *p*. $\phi \left(x\right)=\sqrt{x^{2}+\varepsilon }$. Using the gradient descent algorithm to minimize the objective function, the spatio-temporal coherence of the image without shadow can be obtained.

### 2.6. Network structure and training

The proposed network structure adopts encoder-decoder structure of U-Net (Lee et al., 2022). U-Net structure consists of a contraction channel and an expansion channel. The contraction channel is used to extract contextual features, while the expansion channel is used for image up-sampling to obtain a generated image. The discriminator of the proposed network is also consistent with Lata et al. (2019), it contains multiple convolution blocks. The convolution layer is followed by Batch Normalization and activation function LeakyRelu. The last layer of the discriminator is a Sigmoid function, which outputs the probability value of the true image. In the training process, the generation network and the discriminant network are optimized by the alternating gradient updating strategy. First, the boundary and entropy discriminant networks are optimized to minimize the objective function. Second, the generator network, generation loss, and hierarchical domain adaptation loss are optimized. The overall loss function of the generator network is shown in Equation (17):


(17) $$\begin{array}{l} L=L_{M}+L_{G_{b}}+L_{G_{m}} \end{array}$$

**Figure 6:**
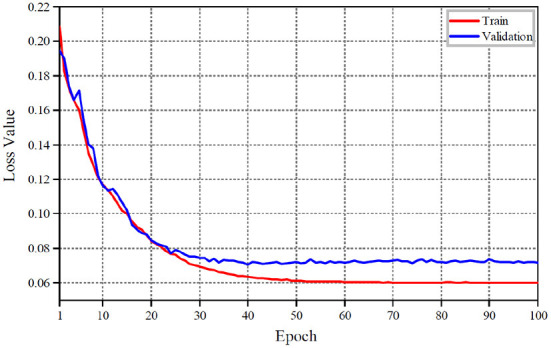
Loss value curves.

**Figure 7:**
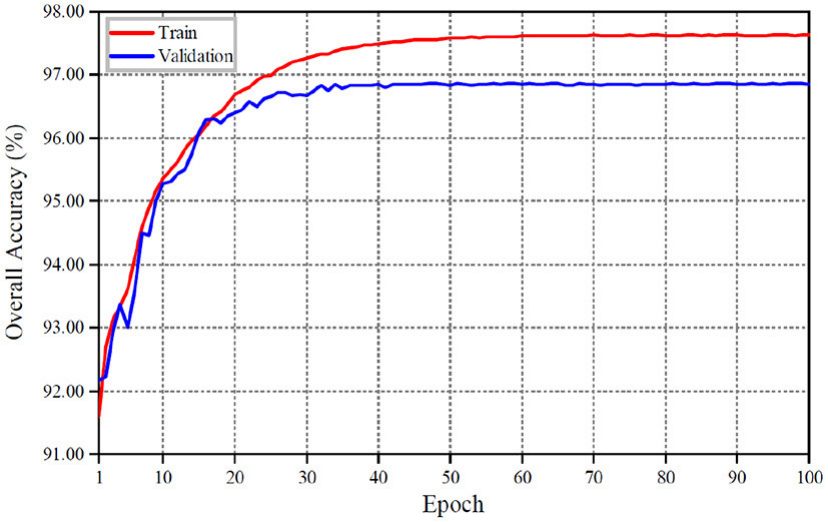
Overall accuracy curves.

The detailed variations of the overall loss value and its accuracy at each training stage are shown in Figures 6, 7 with our proposed shadow detection method. As can be seen from Figures 6, 7, the convergence process of the proposed method is stable, which reduces the over-fitting phenomenon effectively. The overall accuracy exceeds 96%.

## 3. Experiments and analysis

**Figure 8:**
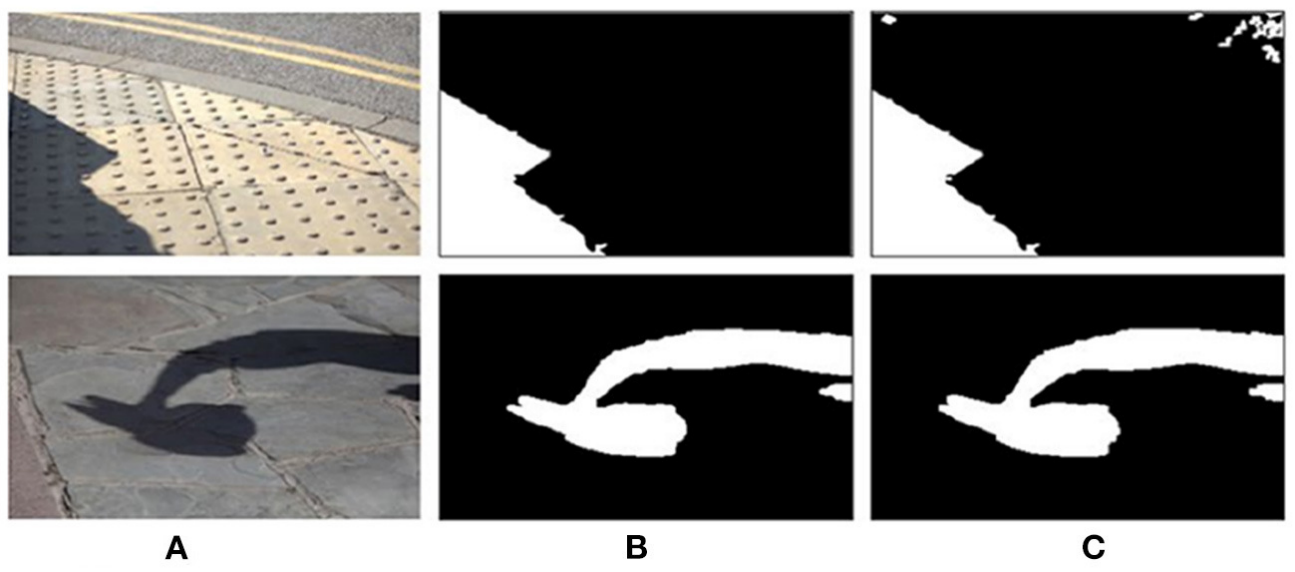
**(A–C)** Datasets for network training.

The experiment platform is: Python programming language, TensorFlow package, Ubuntu 18.04, 16 GB memory, Inter i7 CPU, and NVIDIA GTX1060TI. In the network, the slope of LRELU is set to 0.25, and the objective function is optimized by Adam. The 286 × 286 pixel image in the data set is cropped into 256 × 256 pixel sub-images and flipped to increase the training data. λ_1_ = λ_2_ = 0.5. The initial learning rate is 0.1. As shown in Figure 8, three groups of different training images in the source data set are shown. The three groups of images represent simple geometric boundary shadow, text mixed shadow and complex structure shadow image, respectively. The training data sets with various scenarios are more conducive to the generalization of the network model.

The proposed method is compared with three new shadow detection methods: GSCA-UNet (Jin et al., 2020), SAS (Fan et al., 2020), DSAN (Li et al., 2020). GSCA-UNet aimed to generate additional shadow images to enhance the generalization ability of the model. SAS was constructed based on two CGANs. The multi-task learning mode was used to perform shadow detection and shadow removal tasks successively. DSAN preserved the semantic information of each convolution layer by changing the network connection in the encoding and decoding process to improve the accuracy of shadow detection based on the traditional U-Net image generation model. They are tested on ISTD dataset.

**Figure 9:**
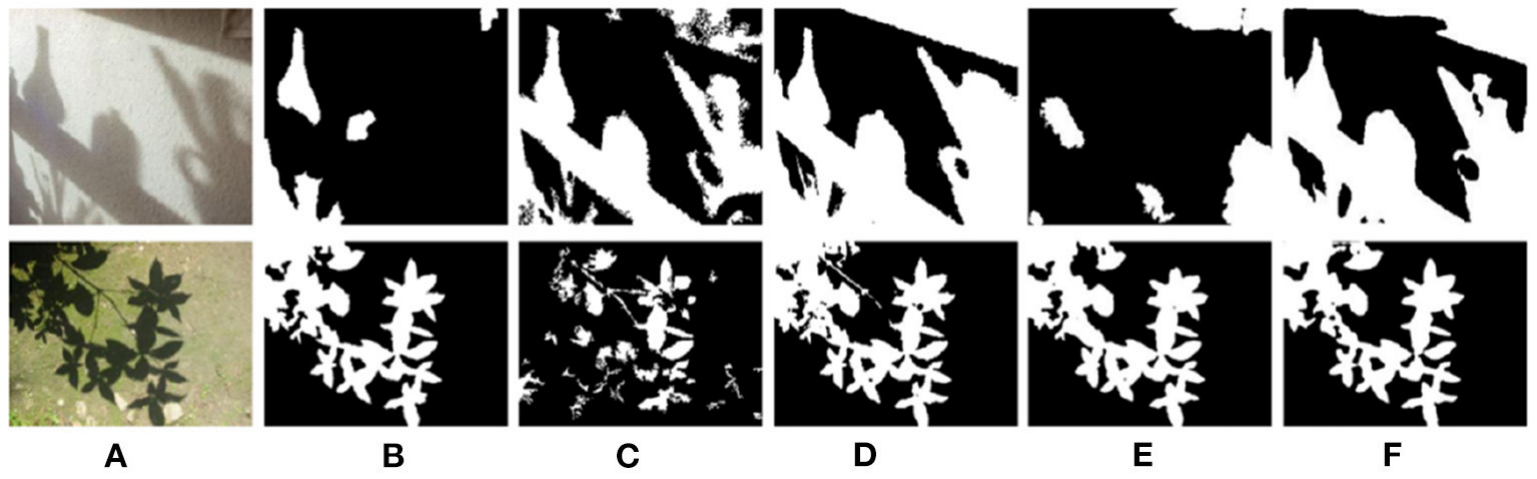
Detection results with different methods on ISTD dataset. **(A)** Shadow image, **(B)** GT, **(C)** GSCA-Unet, **(D)** SAS, **(E)** DSAN, and **(F)** Proposed.

Figure 9 shows the shadow detection effect of different methods in four different shadow scenes. By comparing Figures 9C–F, it can be found that the entropy-driven adversarial learning model also has a great performance improvement in the source domain. In the complex shadow scenes, such as cross texture, text confusion, and irregular shape, it can also get better detection results and has better robustness. It is worth noting that, compared with the incomplete shadow detection results in DSAN, the necessity of boundary adversarial branch can also be reflected in a lateral way.

**Figure 10:**
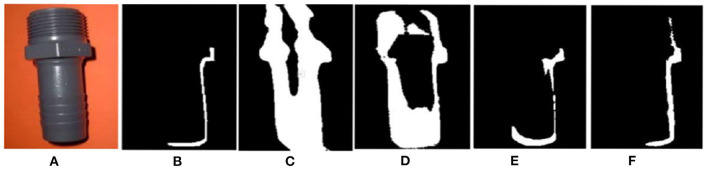
Detection results with different methods on SBU dataset. **(A)** Shadow image, **(B)** GT, **(C)** GSCA-Unet, **(D)** SAS, **(E)** DSAN, and **(F)** proposed.

In order to verify the cross-domain detection performance of the proposed method, a cross-domain comparative experiment is conducted between the proposed method and references (Chen et al., 2018; Shafiq et al., 2020c; Zhang et al., 2020) on the SBU dataset. Figure 10 shows the detection performance of different methods in four different shadow scenes. In the first line, due to the combination of multi-layer domain adaptive feature extraction process, the proposed method will not mistake the black shorts of athletes as shadows. Similarly, compared with the other two methods, the proposed method also has better accuracy in the soft shadow images in the third and fourth rows. In this paper, boundary adversarial branch and entropy adversarial branch are combined, so that the shadow detection results have good boundary structure, and the shadow boundary is smooth and natural.

**Figure 11:**
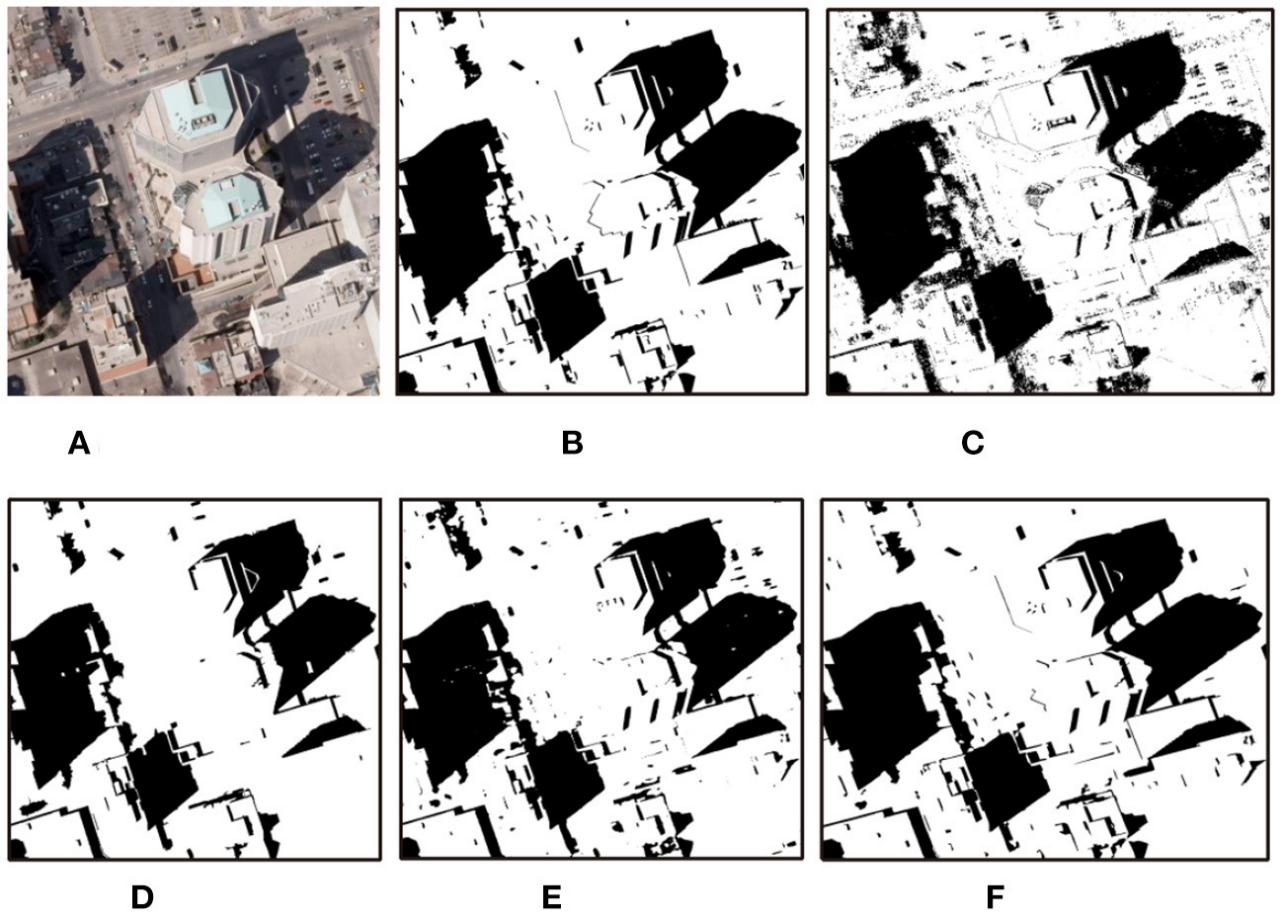
Detection results with different methods on remote sensing images (Toronto). **(A)** Shadow image, **(B)** GT, **(C)** GSCA-Unet, **(D)** SAS, **(E)** DSAN, and **(F)** proposed.

**Figure 12:**
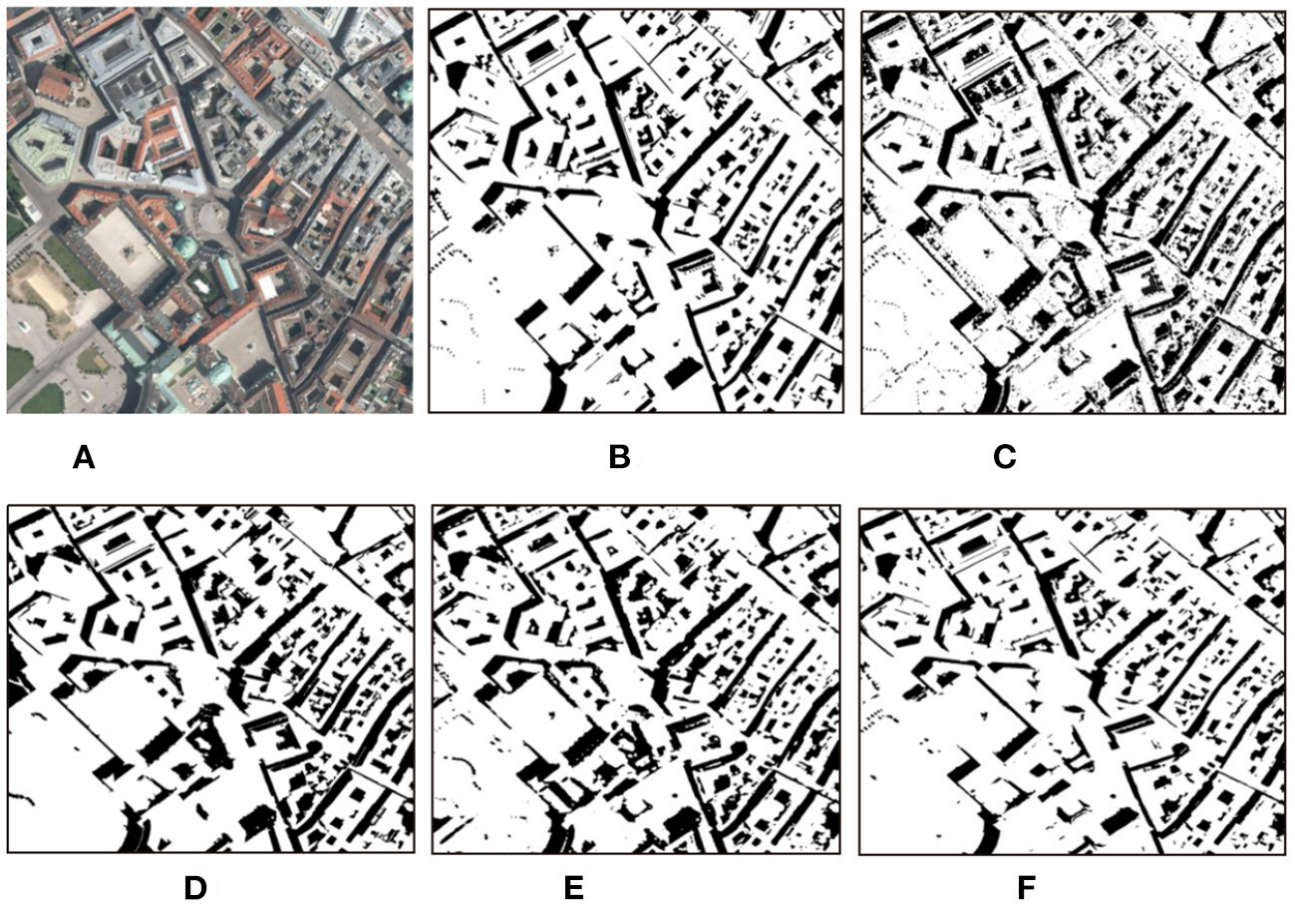
Detection results with different methods on remote sensing images (Vienna). **(A)** Shadow image, **(B)** GT, **(C)** GSCA-Unet, **(D)** SAS, **(E)** DSAN, and **(F)** proposed.

We also conduct experiments on some remote sensing images, the results are shown in Figures 11, 12.

The obtained results by the GSCA-UNet method look reasonable compared to the GT maps. However, as shown in Figures 11C, 12C, quite a few positive (shadow) and negative (non-shadow) samples are needed to be labeled in advance to yield the final detection results for each input image. Another obvious weakness of the GSCA-UNet method can be found from Figures 11C, 12C, where some small shadows are missed, which is caused by the fact that it is arduous to mark small shadows. The SAS method maintains well the integrity of the detected shadow regions, as illustrated in Figures 11E, 12E. Unfortunately, the SAS method fails to handle the nonuniform shadows in the Toronto image and the dark water body in the Austin image. The DSAN method produces satisfactory detection results. However, it can be seen in Figure 11 that it is insufficient to precisely locate the shadow boundaries (Shafiq et al., 2022), and part of the shadows was missed due to lack of consideration for global spatial contextual information. From the aforementioned comparisons, we can conclude that the balance between automaticity and accuracy for the proposed method is better than that of other advanced methods.

We select three evaluation indexes Root Mean Squared Error (RMSE), Balance Error Rate (BER), and Per pixel Error Rate (PER) to evaluate the proposed method.


(18) $$\begin{array}{l} BER=1-0.5\left(\frac{TP}{TP+FN}+\frac{TN}{TN+FP}\right) \end{array}$$

where *TP, TN, FP*, and *FN* are the correctly detected shadow pixels, correctly detected non-shadow pixels, wrongly detected shadow pixels and wrongly detected non-shadow pixels, respectively.

Table 1 shows the same domain detection analysis on the ISTD dataset. Table 2 shows the cross-domain detection analysis on the SBU dataset. Table 3 shows the detection results of remote sensing images. It can be seen that the proposed method is better than other methods.

**Table 1:** The average detection results on the ISTD dataset.

**Method**	**RMSE**	**BER**	**Shadow (PER)**	**Non-shadow (PER)**
GSCA-UNet	14.7	8.5	7.9	7.2
SAS	13.8	7.2	7.2	6.4
DSAN	12.3	6.9	7.1	5.8
Proposed	9.6	6.6	6.9	5.1

**Table 2:** The average detection results on the SBU dataset.

**Method**	**RMSE**	**BER**	**Shadow (PER)**	**Non-shadow (PER)**
GSCA-UNet	14.5	10.2	10.2	11.1
SAS	12.2	9.8	9.7	9.3
DSAN	11.9	8.7	8.8	8.6
Proposed	10.3	7.6	7.1	7.7

**Table 3:** The average detection results on the remote sensing dataset.

**Method**	**RMSE**	**BER**	**Shadow (PER)**	**Non-shadow (PER)**
GSCA-UNet	13.7	10.7	11.7	12.3
SAS	11.9	9.2	10.8	9.7
DSAN	10.2	8.4	8.9	8.8
Proposed	9.8	8.1	7.4	8.2

Table 4 is the computation time comparison, which also shows the better effect with the proposed method.

**Table 4:** The average detection time with different methods.

**Method**	**Time**
GSCA-UNet	6.7
SAS	3.8
DSAN	2.3
Proposed	1.2

## 4. Conclusion

Because existing shadow detection methods only have good performance on source data sets, a novel shadow detection method is proposed in this paper. The method aims to obtain the same accurate detection results on the target data set as on the source data set. Firstly, combined with the hierarchical domain adaptive feature extraction method, a domain classifier is added after each convolution layer in feature extraction process to reduce the data differences between domains, and thus it improves the robustness of the model. Secondly, boundary adversarial branch and entropy adversarial branch are used to obtain smooth boundary detection results. Compared with the most advanced shadow detection methods, the proposed method not only has a great improvement in the source domain, but also has advantages in the target domain. In the future research work, we will consider to further improve the performance of the model from the perspective of generating diverse shadow image data.

## Data availability statement

The original contributions presented in the study are included in the article/supplementary material, further inquiries can be directed to the corresponding authors.

## Author contributions

All authors listed have made a substantial, direct, and intellectual contribution to the work and approved it for publication.

## Funding

Key Scientific Research Project of Higher Education in Henan Province, Teaching Science and Technology [2021] No. 383. Project Number: 22B510017. Project name: Indoor positioning research of quadrotor UAV based on MULTI-sensor fusion SLAM algorithm.

## Conflict of interest

The authors declare that the research was conducted in the absence of any commercial or financial relationships that could be construed as a potential conflict of interest.

## Publisher's note

All claims expressed in this article are solely those of the authors and do not necessarily represent those of their affiliated organizations, or those of the publisher, the editors and the reviewers. Any product that may be evaluated in this article, or claim that may be made by its manufacturer, is not guaranteed or endorsed by the publisher.
